# Understanding suicide

**DOI:** 10.15190/d.2024.2

**Published:** 2024-03-31

**Authors:** Lavinia Horoșan, Diana-Elena Nistor, Adriana Ion, Mihai Corban, Ana Giurgiuca

**Affiliations:** ^1^Clinical Hospital of Psychiatry Professor Doctor Alexandru Obregia, Bucharest, Romania; ^2^University of Medicine and Pharmacy Carol Davila, Bucharest, Romania

**Keywords:** Suicide, psychiatry, mental health, neurobiology, prevention.

## Abstract

Suicide remains a significant public health challenge globally, requiring comprehensive approaches for prevention and treatment. Almost 90% of individuals who commit suicide suffer from mental health disorders at the time of death, emphasizing the central role of psychiatry in understanding and preventing suicide. Suicidal thoughts, planning, attempts, and completed suicides exist on a continuum, with the highest suicide rates occurring within six months after an attempt and more severe attempts increasing the risk of future suicide. History plays a significant role in shaping the perception of suicide, from considering it a sin to recognizing it as a mental illness. Emile Durkheim's ground-breaking work on suicide as a social phenomenon furthered our understanding. Knowledge regarding the complexities surrounding suicide is paramount to developing effective strategies. Early detection through clinical interviews and screening tools and creating safe spaces for discussion are critical prevention measures. Pharmacological and non-pharmacological interventions are essential for addressing underlying psychiatric disorders and reducing suicidal thoughts and behaviors. While significant progress has been made in understanding suicide risk factors and implementing prevention strategies, continued research and community engagement are imperative. Destigmatizing mental health discourse and fostering supportive environments are essential steps toward reducing the incidence of suicide and supporting individuals in distress. By embracing a holistic approach that integrates pharmacological and non- pharmacological interventions, along with societal engagement, we can strive towards a future where suicide is increasingly rare, and individuals feel valued, supported, and connected.

## SUMMARY

*1.* *Introduction*

*2.* *History*

*3.* *Epidemiology*

*4.* *The neurobiology of suicide*

*5.* *Suicide and mental health disorders*

*6.* *Treatment*

- *6.1.* *Prevention*

- *6.2.* *Pharmacological treatment*

- *6.3.* *Non-pharmacological treatment*

*7.* *Conclusion*

## 1. INTRODUCTION

Suicide is a complex phenomenon, and yet not fully understood, with no single cause and multiple domains of risk factors and valid perspectives for both scientific explanation and prevention. Among people who commit suicide, a great majority, representing almost 90%, usually suffer from multiple mental health disorders at the time of death, placing the act of suicide as a central factor for psychiatry, being responsible for about 1% of all deaths worldwide^1-3^. Suicidal thoughts, planning, attempts and completed suicides represent a continuum of suicidal behaviours. Suicide rates are highest during the first six months after an attempt, while violent and more severe attempts further increase the risk of future suicide, multiple attempts being correlated with eventual completed suicide^4^.

Suicide can occur at any age and it is the second most frequent, in some countries even the first, cause of death among individuals aged 15 to 24. What we know now is that suicide is multifactorial, occurring from the convergence of genetic, psychological, social and cultural risk factors combined with experiences of trauma and loss. Research on suicide is highly challenging because of its complexity as a multifaceted phenomenon. However, the recognition of suicide prevention as a public health priority and national prevention programmes have encouraged research, detection, treatment, and management of people at risk for suicide in many countries^5,6^.

A great emphasis was built around any therapeutic interventions that can be of value when it comes to suicide ideation, and both pharmacological and non- pharmacological interventions are continuously reviewed with the ultimate purpose of elaborating a better answer to this problem. A list of risk and protective factors split into individual-level and social-level was elaborated with the purpose of ultimately identifying individuals that are at risk and elaborating a complex strategic and individualized approach based on these factors. Among protective factors, we find on a personal level: problem-solving skills, increased tolerance to frustration, self-control, reasons for living and optimism, perceptions of positive health, participating in sports activities; and on a social level: family relationships and partnerships, social relationship and social support, religious and spiritual beliefs and employment. On the other hand, we can enumerate a series of risk factors both on an individual level (such as prior suicide attempts, mental disorders, trauma or abuse history, hopelessness, stressful life events, self-harm, prior psychiatric hospitalization, family history of suicide, chronic illness or pain, personality traits, biomedical or physical determinants) and on a social level (for example job or financial loss, socio- economic disadvantage, relationship conflict, discord or loss, disaster, war or conflict, acculturation stress)^6-8^.

For the population at risk, suicide is one of the most disturbing events that can happen to a human being, and, moreover, it implies a lot of pain often shared by relatives and persons who were close. It has been demonstrated that the loss of a person to suicide may be one of the most distressing events that may occur in mental health professionals, resulting in several negative consequences, such as lower quality of life and work productivity^9^.

## 2. HISTORY

Suicide and mood disorders are one of the main domains of interest when it comes to psychiatric history that leads back to more than 2000 years ago, being described by Hippocrates in 460-377 BC as symptoms of melancholia. Although the diagnostic and therapeutic measures, especially terminology, come from a long history, many controversies remain, and a significant part of the medical management is unsatisfactory. In ancient Greece, Egypt and Rome, suicide was regarded as a sin and people were abandoned in the streets and left to the animals. Only suicides in which it was possible to find sufficient reason for self-destruction were deemed to be comprehensible, some examples including heroism, love-rejection or severe and painful illness. Other reasons were considered unjustified, and they were punished^10^. Only later, around 1600, in the United Kingdom, suicide was first seen as a mental illness^11,12^. Possibly the first recorded suicide dates back to the era of the Egyptian Pharaoh Ramses II (1303- 1213 BC), in a written description of two brothers who had committed suicide nearly two centuries previously^10^.

Emile Durkheim, a French philosopher, intensively studied the phenomena of suicide and postulated two core principles, seeing suicide no longer as a sin but a result of a more complex and elaborated cumulation of the concepts that society impregnates on an individual. At the time he published his book, *Le suicide, *suicide was seen as a psychological issue. He postulated that the structure of suicide rates is a positive function of the structure of a group or a class of people's social relationships and that social relationships vary according to their level of integration and moral regulation. He defined four types of suicide^13-16^ (Table 1).

**Table 1:** Table 1. Types of suicide according to Emile Durkheim

Type of suicide	Description
Egoistic	Occurs when the individual does not feel as if they are well integrated into society or as part of the community, but the community also feels like the individual is not a part of their lifestyle. Because of the lack of moral support to get through the difficult parts of life and the feeling of abandonment, suicide is then seen as an answer to end the carousel of bad emotions that are not contained in any supportive community^13-16^
Altruistic	As opposed to the egoistic dimension of suicide, the altruistic one occurs when the individual is too integrated into society and the pressure to succeed for the greater good becomes overwhelming. Here, it is described as the kind of behaviour resulting from the need to become part of a community, up to the point that his own life can be dedicated to this. As an example, here we remind of suicide bombers or of kamikaze^13-16^
Anomic	Is likely to occur when the regulation of society is disrupted, for example, during an economic boom or depression, that leaves individuals out of guidance over the norms and values that they should be following. In this scenario, people no longer understand which norms and values still apply and that new norms need to be followed. Because of the lack of regulation, people remain overwhelmed by the changes and the lack of predictability^13-16^.
Fatalistic	Occurs when the norms of the society are excessive, an example being the social restriction that was imposed on slaves. People who have a sense of lack of control over their lives are more prone to commit suicide^13-16^.

After Durkheim, multiple theories arose, trying to complete and find better explanations for the multiple questions that surround death by suicide. In 1954, Henry and Short postulated that violent aggression resulting from increased frustration is the source of this behaviour. If the violent act is oriented towards people around, it would result in homicide, and if it is oriented towards oneself, it results in death by suicide^17^. Later, in 1964, Gibbs and Martin postulated that a conflict between social roles related to age, sex, occupation and marital status were crucial factors in determining suicide, meaning that in situations when individuals experienced a high degree of irresolvable conflict, suicide arose as a possible solution^17,18^.

Multiple theories arose over time, trying to incorporate all factors that are implicated into the complex act of suicide. Among them, the stress-diathesis model depicts suicidal behaviour as a consequence of an interaction between acute stressors or proximal risk factors and a diathesis or distal factors. Stressors can be external (for example, financial or relationship problems) or internal (for example, a major depressive episode)^19,20^.

The effort to explain suicide as a neurobiological phenomenon was directed by the fact that a great proportion of individuals committing suicide had a comorbid psychiatric disorder. Analyzing post-mortem the brains of suicide victims in search of neurotransmitter-related abnormalities, including serotoninergic, dopaminergic, noradrenergic, signal transduction and cellular morphology, had encountered both difficulties, such as a complete psychological evaluation, and advantages, such as the fact that individuals who have completed suicide attempts are the most severe cases and therefore with the most likely neurobiological implications. One of the most influential theories that arose from those studies was the serotoninergic hypothesis that reflected a dysfunction in the serotoninergic function in the prefrontal cortex, hippocampus and occipital cortex, represented by fewer serotoninergic synapses and fewer presynaptic serotonin transporter sites. Less studies as for the serotoninergic system were conducted regarding both noradrenergic and dopaminergic systems. Findings suggest that there are fewer noradrenergic neurons in the locus coeruleus in suicide victims with major depression, but non-specific illness or stress effects cannot be ruled out. In addition, noradrenaline levels seem to be lower in the brainstem of suicide victims, whereas α2 -adrenergic receptor numbers are higher, perhaps upregulated secondary to lower noradrenaline levels^21-23^.

## 3. EPIDEMIOLOGY

Suicide is one of the leading causes of death among young, respectively the 4th among 15 and 29-year-olds globally in 2019. Every year, 703,000 individuals take their own life, and there are many more who attempt suicide. This phenomenon does not occur only in high-income countries, but in all regions around the globe. Over 77% of suicides worldwide occur in low- and middle-income countries, according to the World Health Organization (WHO) in 2019^6^.

According to data from the Substance Abuse and Mental Health Services Administration (SAMHSA) in the United States for the year 2021, approximately 12.3 million adults reported experiencing serious suicidal ideation, with 3.5 million adults formulating a plan, and 1.7 million adults attempting suicide. This resulted in 48,183 recorded deaths by suicide, equating to approximately one death every 11 minutes in the United States during that year^24^.

According to the Centers for Disease Control and Prevention (CDC) provisional data, there is a 2.6% increase in overall suicide deaths in the United States from 2021 to 2022. While there was a slight increase in suicide deaths among males (2.3%) and females (3.8%), there were notable variations in suicide rates across different racial/ethnic groups. Specifically, there was a decrease in suicide deaths among American Indian or Alaska Native individuals (6.1%), while increases were observed among Asian (5.7%), Black or African American (3.6%), Native Hawaiian or Other Pacific Islander (15.9%), White (2.1%), Multiracial (7.9%), and Hispanic or Latino (4.3%) populations. Regarding age groups, there was a decrease in suicide deaths among individuals aged 10-24 years (-8.4%), while slight increases were noted among those aged 25-44 years (0.7%), 45-64 years (6.6%), and ≥ 65 years (8.1%)^25^.

WHO estimates that about 20% of global suicides are due to self-poisoning with pesticides, most of them occurring in rural agricultural areas in low- and middle-income areas. Among other common methods, hanging and firearms are listed^26^. Women also commit suicide using poisoning at a rate of 31%^27^. In the Asian region, the most frequent method is hanging (at rates as high as 69% in Japan), except for Hong Kong, where people most often end their lives through falls (approximately 43%)^28^. In Europe, males most commonly commit suicide by hanging (up to 91% in Poland), except Swiss males who use firearms, and females more commonly use hanging and poisoning^29,30^.

Data on deaths by suspected suicide in England between October 2022 and December 2023 suggests a general raise in rates among female population, coupled with a decline in the entire 45 to 64 age brackets in recent months. The proportion of deaths for method strangulation, hanging and suffocation is consistently the highest across all quarters, however it has shown a continual decrease across the reporting period, unlike poisoning and drowning that showed an increase^31^. The overall findings suggest that men more often complete suicide attempts compared to women, but the numbers vary across different countries, with the highest rates for women found in China, Angola, Japan and Belarus, compared to Kazakhstan, Mongolia, Lithuania and Sri Lanka who reported higher rates for suicide in men^30,32^.

## 4. THE NEUROBIOLOGY OF SUICIDE

At the heart of numerous models lies a stress- diathesis element, suggesting that suicidal behavior arises from the interplay of acute stressors and a predisposition towards suicidal behavior (the diathesis)^23^.

One hypothesis suggests that suicidal behavior results from multiple subtypes. This theory posits two main patterns of suicidal thinking: stress-responsive and non-stress-responsive. In the stress-responsive pattern, individuals experience sudden increases in suicidal thoughts after stressful events, leading to impulsive suicidal actions. This pattern may be linked to childhood trauma, associated with impulsive aggression among adults, and disruptions in serotonin and hypothalamic-pituitary axis functions. Conversely, the non-stress-responsive pattern involves persistent suicidal thoughts and is associated with depressive affect, leading to more carefully planned suicidal behavior. Biomarker studies in depression indicate a neurobiological basis for more carefully planned suicidal acts, with lower cerebrospinal fluid serotonin metabolite levels correlating with increased planning and lethality. Additionally, individuals with a history of carefully planned attempts demonstrate better cognitive control than those with impulsive attempts, implicating executive function and reward circuits in the development of these distinct suicidal subtypes^33^.

The frontal and prefrontal cortex play a vital role in suicidal behavior through their participation in cognitive functions, stress response regulation, and the control of impulsivity. Individuals who have attempted suicide in the past display altered activation patterns in the prefrontal areas, leading to challenges in decision-making, assessing risks and rewards, and social judgment. The anterior cingulate cortex, responsible for processing negative self-thoughts and emotions, is heavily associated with suicidality. In comparison to depressed individuals who have not attempted suicide, those who have attempted suicide exhibit increased activation of the anterior cingulate cortex and orbitofrontal cortex when exposed to emotionally expressive faces, indicating differences in emotional stimulus processing^34-36^.

Psychological tools assess suicidal risk by questioning individuals but often lack predictive accuracy due to patient reluctance and false positives. Exploring biological markers could enhance risk assessment, particularly in severe depression, psychosis, or hospitalization contexts^37^.

### Inflammation and kynurenine pathway

The etiology of suicide appears to be linked with neuroinflammation, which triggers the kynurenine pathway, leading to serotonin depletion through tryptophan consumption, the precursor of serotonin. Proinflammatory cytokines like interferon-γ (IFN-γ), IL-1β, and IL-6 activate this pathway by stimulating enzymes such as indoleamine 2,3 dioxygenase (IDO) and tryptophan 2,3-dioxygenase (TDO). Further metabolism along this pathway generates neuroactive compounds like quinolinic acid (QUIN) and kynurenic acid (KYNA), affecting glutamatergic neurotransmission. QUIN, an NMDA agonist, with its excitotoxic and proinflammatory properties, may contribute to neuronal loss and reduced hippocampal volume. Increased QUIN levels have been observed in the cerebrospinal fluid (CSF) of suicide attempters and those with suicidal intent, irrespective of mood disorder comorbidity. Conversely, the neuroprotective picolinic acid (PIC) levels were reduced in the CSF and blood of suicide attempters. Furthermore, suicide attempters exhibited a decreased PIC/QUIN ratio in their CSF and blood, with sustained reduction of PIC in the CSF for up to two years post-attempt^37-39^.

### BDNF

Abnormal neuroplasticity is a distinguishing feature among suicidal patients. Studies reveal reduced mRNA levels of BDNF and its receptor TrkB in the prefrontal cortex and hippocampus of individuals who commit suicide. Additionally, depressed patients with suicidal inclinations display decreased BDNF expression in the anterior cingulate cortex and amygdala, as well as lower levels of BDNF in the serum, compared to depressed patients who do not have suicidal intentions. Some data suggest that serum BDNF could serve as a promising marker, indicating its potential utility, while others deny this finding^37^.

### Serotonin

Serotonin deficiencies are linked to the development of depression and are also associated with increased tendencies towards aggression, impulsivity, suicidal ideation, and attempted suicide. In individuals who have attempted suicide with greater lethality, cerebrospinal fluid (CSF) concentrations of the primary serotonin metabolite, 5-hydroxyindoleacetic acid (5-HIAA), are diminished, serving as a prognostic indicator for future suicide risk. The majority of investigations into suicide victims have noted a reduced density of serotonin transporter in the prefrontal cortex, anterior cingulate, and hypothalamus. However, there are conflicting findings from some studies which report no alterations in these regions. Most studies suggest that individuals displaying suicidal tendencies commonly show heightened expression of 5HT1A and 5HT2A receptors in both the raphe and prefrontal cortex. This elevation in receptor expression may serve as a compensatory response to diminished serotoninergic neuron activity. Nevertheless, conflicting results emerge, as certain studies report no differences or even a decrease in the expression of these receptors^37,40,41^.

### The Hypothalamic-Pituitary-Adrenal Axis

Suicidal behaviors appear to be linked with overactivity of the HPA axis, potentially resulting in disrupted stress regulation, compromised hippocampal function, and cognitive impairment. Adrenal gland cortical hypertrophy was observed in individuals who died by suicide. Elevated cortisol levels in saliva, CSF, and plasma have been documented in individuals who have attempted suicide compared to those in healthy volunteers. Studies examining depressed individuals who have died by suicide compared to nonpsychiatric control subjects indicate elevated levels of corticotropin- releasing hormone (CRH) and vasopressin in the forebrain, raphe, and locus coeruleus. Furthermore, fewer CRH receptors type 1 (CRHR1) are observed in the frontal cortex, likely indicating down-regulation in response to heightened CRH levels. There is also an increase in proopiomelanocortin (POMC) in the pituitary gland, along with decreased expression of glucocorticoid receptors (GR) in the amygdala and prefrontal cortex, although not in the hippocampus. Additionally, there is a decrease in mRNA expression of hippocampal mineralocorticoid receptors (MR), mirroring patterns observed in rodent models of chronic stress^37,41,42^.

### Norepinephrine

Based on numerous observations, it has been suggested that dysfunction in catecholaminergic systems may contribute to suicide. Suicide victims have been noted to exhibit elevated levels of norepinephrine (NE) alongside reduced alpha2- adrenergic receptor binding in the prefrontal cortex. Increased levels of norepinephrine have been linked to elevated aggression levels. Likewise, diminished norepinephrine levels seem to mitigate the impact of childhood abuse on the emergence of aggressive behaviors or impulsivity during adulthood in males^43^.

### Glutamatergic and GABAergic Neurotransmission

The efficacy of glutaminergic NMDA receptor antagonists, such as ketamine/esketamine, in reducing suicide rates indicate the potential involvement of glutamate in this mechanism. Current evidence suggests that glutamate may exert a significant influence on suicide-related personality traits, such as impulsivity and aggression. This occurrence might be linked to heightened levels of the NMDA receptor agonist QUIN in the central nervous system of individuals who have attempted suicide, stemming from the activation of the kynurenine pathway as a result of IDO stimulation by proinflammatory cytokines. Following this, the majority of studies have indicated reductions or the absence of differences in NMDA binding in the prefrontal cortex among individuals who have died by suicide. There is evidence of a disrupted balance between glutaminergic and GABAergic neurotransmission in suicide risk, though research shows discrepancies^37^. Structural MRI observations primarily reveal deficits in grey matter volumes within cortical regions (such as the orbitofrontal, dorsolateral prefrontal, insula, and superior temporal gyrus) and basal ganglia (including the caudate and globus pallidus), predominantly on the right side. Notably, suicide attempters tend to exhibit larger volumes of the thalamus and right amygdala. Moreover, findings indicate the presence of white-matter hyperintensities, particularly in periventricular areas, along with increased bilateral volumes of inferior frontal white-matter tracts, notably the uncinate fasciculus and inferior orbitofrontal fasciculus. Additionally, reduced anisotropy is observed in the left orbitofrontal area and the left anterior limb of the internal capsule, suggesting structural connectivity impairments associated with suicidal behavior. Functional neuroimaging studies related to suicidal behavior reveal altered reactivity to various stimuli, primarily observed in the bilateral orbitofrontal cortex, right ventromedial and anterior cingulate cortex, and left dorsolateral prefrontal cortex regions. Additionally, there is a decrease in functional connectivity between the anterior cingulate and posterior insula, while connectivity increases in a striatal motor-sensory network^44^.

## 5. SUICIDE AND MENTAL HEALTH DISORDERS

A large majority of suicides and suicide attempts are related to a mental health disorder, with a reported percentage going as high as 98%. The remaining percentage is represented by suicides that occur in relation to financial or relationship problems and corresponding crises. Discrimination, violence, terror and war represent other causes.

Having an overall look, at the beginning of the 21st century, the highest mortality of unnatural causes globally was due to depression (30%), followed by substance-use disorders (18%), schizophrenia (14%) and personality disorders (13%). It is important to note that there is a difference between in- and outpatients. When we look at the inpatient profile, almost 45% were due to schizophrenia and organic mental disorders, and, for the outpatients, 32% occurred in the context of depression, substance-related disorders, somatoform, anxiety and adjustment disorders. Depression is one of the most important diagnostics, being present in both groups, but with different rates^30,45,46^. Furthermore, during the COVID-19 pandemic, social isolation intensified mood disorders, such as depression and anxiety, underscoring the impact of environmental influences on symptom manifestation^47^.

Two systematic reviews with meta-analyses shed light on the impact of the COVID-19 pandemic on suicide-related outcomes. Y. Yan and colleagues found an increase in suicidal ideation and suicide attempts, despite the stable suicide rate, underscoring the pressing demand for prevention and intervention programs. Similarly, J. Dube et al. reported elevated rates of suicide ideation, suicide attempts, and self- harm during the pandemic compared to pre-pandemic levels^48,49^.

Special attention must be paid during the next 4 to 12 weeks after the discharge from the psychiatric ward, when suicide rates rise, especially in males and in individuals with a history of suicide attempts^50,51^.

Schizophrenia and psychotic episodes are strongly associated with higher suicide risk, being responsible for about 2% of global deaths, both during the acute episodes and during the inter-episodic period^6,52^. E. Bleuler characterized schizophrenia symptoms - "the suicidal drive", as "the most serious of all schizophrenic symptoms" in 1911. Later, in 1919, Kraepelin stated that suicide happened both in acute and chronic stages of schizophrenia. The lifetime rate of suicide in individuals diagnosed with schizophrenia is between 4 and 13%, with suicide being the most important factor that shortens the lifespan of those patients^53^. A greater risk of suicide when we talk about schizophrenia is seen between the first episodes of the disease when the burden of the diagnostic is higher. Otherwise, risk factors for suicide behavior in schizophrenia are similar to those for the general population. Suicide risk was associated with male gender, higher IQ, agitation of motor restlessness and a personal history of suicide attempts or suicidal ideation. Data showed heterogenous results regarding both positive (delusions and hallucinations, including imperative auditory hallucinations) and negative symptoms impact. Comorbid psychiatric disorders such as depressive episodes and alcohol or psychoactive substance use are strongly associated with suicidal risk^53-55^.

Mood disorders are frequent and recurrent and are associated with a lifetime risk of suicide that is about 15 times higher than in the general population. Bipolar disorder is one of the main causes of disability in young people that leads to cognitive and functional impairment and raised mortality, particularly by suicide^56^. Patients who feature suicidal ideation or have a history of suicide attempts represent the more severe cases. Among mood disorders, mixed states are associated with a higher risk of suicide behavior. Looking at the overall causes of premature mortality in patients with mood disorders, suicide occupies the first place. Therefore, treatment options are built up around suicide prevention, and now we can access both pharmacological and non-pharmacological options^57,58^. Numerous risk factors have been enumerated as being important when we look at the suicide risk in patients with mood disorders, such as sociodemographic and social factors (such as younger age or poor social support), illness-related factors (such as severe or recurrent depression, failure to achieve remission, early age onset, psychotic symptoms, melancholia), other psychiatric comorbidities (such as personality disorders, especially borderline personality disorder, alcohol dependence or misuse), or chronic physical illness^1^.

Substance use disorders represent the second most common reason for suicide in outpatients (22.4%), with the incidence in inpatients being twice as high. In both situations, alcohol is the main substance used, with a percentage as high as 85% of suicides in substance use being related to alcohol and/or sedative-hypnotic drugs. Compared with the general population, those with alcohol use disorders are almost ten times more likely to die by suicide, and those who inject illicit substances are about 14 times more likely to commit suicide^4^. Substance misuse and dependency frequently co-occur with other psychiatric disorders, most frequently with depression and anxiety, but an important aspect to be underlined is the use of, most frequently, alcohol in pathological gamblers, which determines a higher risk for suicide^30,59-61^. Several predisposing and precipitating risk factors, combined with personality traits (such as impulsivity, aggression, pessimism and hopelessness) and mental illnesses, intensify the risk of suicidal behaviors in addiction patients. Among them, we note occupational and financial stressors, recent heavy substance use and intoxication, a history of previous suicide attempts and sexual abuse, and marital and interpersonal relationship disruption. Although when we look at the overall numbers, males commit suicide more frequently than women, when there is a substance use disorder, the association is significantly stronger in women^4^.

A two-stage theoretical framework rooted in prior research proposes that there is a significant correlation between the tendency to act violently toward oneself and aggression directed towards others. The two-stage model suggests that suicide and violence arise from a common aggressive impulse, with other variables determining its direction. The first stage sets the threshold for impulsive aggression leading to action, while the second identifies the target of this aggression. Numerous variables exhibit a differentially influential role in predisposing individuals to the risk of engaging in either self- directed or other-directed violent behaviors. Factors such as depression, hopelessness, numerous life challenges, trait and state anxiety, and a permissive stance toward suicide demonstrate correlations with an elevated susceptibility to suicidal tendencies. On the other hand, impulsivity, legal entanglements, and recent life stressors are associated with an augmented risk of engaging in violent behaviors. Trait anxiety inversely correlates with violence, suggesting it suppresses outward violence while amplifying inward violence^62-65^.

Anger, aggression, and impulsivity are intertwined psychological traits associated with suicide attempts, proposed to be collectively considered as a unified phenotype^66^. Impulsivity, a key concept in personality theories, encompasses behaviors such as impaired self-regulation, poor planning, hasty decision-making, sensation-seeking, risk-taking, response inhibition difficulties, and prioritizing immediate rewards^67^. Recent studies have revealed that trait impulsivity exhibits greater predictive value for suicidality compared to state impulsivity. Furthermore, the association between impulsivity and suicidality demonstrates greater strength in the domain of behavioral impulsivity than cognitive impulsivity^68^.

Borderline Personality Disorder (BPD) is a severe psychological condition characterized by unstable self-concept and emotional dysregulation. Impulsive actions and suicidal tendencies stand out as hallmark traits of BPD^69^. Reports indicate that between 50% to 90% of individuals with BPD have engaged in suicidal acts at some stage, a rate nearly 50 times higher than that of the general population, thereby identifying BPD as a significant psychiatric risk factor for suicide^70,71^. BPD also poses a substantial risk for aggression towards others, given the anger management challenges of the disorder. Supporting this link, more than 70% of individuals with BPD report involvement in violent behavior within the past year^72^. Studies reveal differences between suicidal BPD individuals who die by suicide and those typically seen in acute psychiatric care. The lethality of suicide in BPD stems from the interaction between impulsivity and violent-aggressive characteristics^73^. Attempters with comorbid BPD and Major Depressive Disorder showed more lifetime suicide attempts, earlier onset of first attempt, increased interpersonal triggers, and higher levels of aggression, hostility, and impulsivity compared to those with Major Depressive Disorder only^74^.

## 6. TREATMENT

### 6.1. Prevention

The importance of knowing and understanding better the complex phenomenon of suicide has the ultimate goal of creating better approaches that can help people get through the difficult time. Being such a wide mental health problem, multiple guidelines have been published, with most of the key-points overlapping, but keeping in mind that early detection is a critical prevention strategy^6^. One of the most important factors is that the clinical interview contains questions that cover the subject, the main problem being that, in many cultures, suicide is seen as a sin, and because of that there is a great reticence in speaking about it with family members or friends. Tools like ASQ (Ask Suicide-Screening Questionnaire Toolkit) are simple but quick to apply and very efficient in determining suicidal ideation in patients of all ages^75,76^. Creating a safe space where the patient feels that he can talk about his problems, including suicidal ideation, is a skill that every clinician must acquire, and it can save lives. In no circumstance must the clinician answer himself the question of suicide for the patient in his place^77,78^.

Considering the list of risk and precipitating factors, a list of strategies that prevent suicide have been gathered and includes strengthening economic support, creating a productive environment, improving access to delivery of suicide care, promoting healthy connections, teaching coping and problem-solving skills, identifying and supporting people at risk and lessening harms and preventing future risk^77,79,80^.

Rising awareness about mental health problems and the existence of a telephone number that can offer support are some of the multiple attitudes that have arisen and are available in most countries. The psychiatric treatment includes a holistic approach of psychotherapeutic interventions and pharmacological treatment^26,77^.

### 2.2. Pharmacological treatment

Suicide presents a significant and escalating public health challenge, yet the availability of research guiding the selection of pharmacological interventions, particularly for patients at elevated suicide risk, is notably scarce. Conducting drug trials involving individuals contemplating suicide is both achievable and highly necessary. While pharmacotherapy is frequently employed to address suicide risk, the absence of robust randomized controlled trials may expose patients to unnecessary risks due to the absence of evidence-based treatment decisions^81^.

According to the recommendations from the Practice Guideline for the Assessment and Treatment of Patients With Suicidal Behaviors, issued by the American Psychiatric Association in 2010, while evidence regarding the reduction of suicide rates with antidepressant treatment remains inconclusive, their efficacy in managing acute depressive episodes and providing long-term benefits in patients with severe anxiety or depressive disorders justifies their use in individuals experiencing suicidal thoughts or behaviors. It is advised to select antidepressants with a low risk of acute overdose lethality, such as selective serotonin reuptake inhibitors, and to prescribe them conservatively, especially for less familiar patients. For individuals with significant insomnia, the consideration of sedating antidepressants or adjunctive hypnotic agents is recommended. Furthermore, benzodiazepines may be indicated for short-term treatment of severe symptoms like insomnia, agitation, panic attacks, or anxiety, with preference given to long-acting agents over short-acting ones. Additionally, sedative medications such as trazodone, low doses of second- generation antipsychotics, or certain anticonvulsants may be considered for highly anxious and agitated patients. Long-term maintenance treatment with lithium salts is strongly associated with major reductions in suicide risk for patients with bipolar disorder and recurrent major depressive disorder. However, there is no clear evidence that specific anticonvulsants alter rates of suicide or suicidal behaviors. Clozapine treatment is linked with significant decreases in suicide attempts and possibly suicide rates for individuals with schizophrenia and schizoaffective disorder. Thus, serious consideration should be given to clozapine treatment for psychotic patients with frequent suicidal ideation or attempts, although potential adverse effects must be weighed. Electroconvulsive therapy (ECT) is recommended for severe depressive episodes accompanied by suicidal thoughts or behaviors, particularly for patients with catatonic features or those in whom delayed treatment response poses a life-threatening risk. However, continuation or maintenance treatment with pharmacotherapy or ECT is advised following an acute ECT course due to the lack of evidence for long- term suicide risk reduction^82^. The European Psychiatric Association guidance on suicide treatment and prevention from 2012 highlights that the clinical prevention of suicidal behaviors is achieved by addressing the underlying psychiatric disorders and targeting specific psychiatric symptoms through treatment^83^.

A systematic review conducted by Masdrakis and Baldwin published in 2023 mentions that the well- documented effectiveness of clozapine in reducing suicide risk among patients with schizophrenia or schizoaffective disorder is widely recognized. While there is some suggestion that it could be beneficial in managing severe and treatment-resistant cases of suicidal ideation and non-suicidal self-injury in individuals with bipolar disorder or borderline personality disorder, the extent and reliability of supporting evidence remain constrained^84^.

H. Reeves et al. conducted a pilot study aiming to explore the efficacy of risperidone augmentation to antidepressants in managing suicidality and core symptoms in major depressive disorder (MDD). MDD poses a significant risk of suicidality, and antidepressant treatment alone may not suffice for managing acute symptoms of depression. The study enrolled twenty-four adults diagnosed with MDD experiencing a depressive episode with suicidality despite antidepressant therapy. The double-blind, placebo-controlled study spanned eight weeks, with subjects randomly assigned to receive risperidone (0.25-2 mg/day) or placebo while continuing antidepressant therapy. Clinical efficacy in suicidality, depressive symptoms, and impulsivity was assessed at various intervals throughout the study. The results indicated that risperidone significantly reduced suicidal ideation in MDD patients, showing superiority over placebo. The onset of risperidone's effect was rapid, observed at two weeks of treatment, and sustained over the eight weeks. Additionally, risperidone demonstrated effectiveness in improving other symptoms related to suicidality and exhibited a higher trial completion rate compared to placebo. Moreover, the subjects tolerated the low dose of risperidone well. In conclusion, findings from this pilot study suggest that risperidone augmentation is beneficial in managing high-risk suicidal ideation in MDD patients experiencing a depressive episode. The rapid and sustained anti-suicidal effect of risperidone is particularly advantageous for the acute management of severe depressive symptoms. Despite limitations in sample size, these promising results warrant further investigation on a larger scale to assess the efficacy of atypical antipsychotics in treating severe depression with suicidality^85^.

In a study focusing on patients aged 60 years and older with treatment-resistant depression, those who did not respond to venlafaxine were randomly assigned to receive either aripiprazole augmentation or a placebo. Subgroup analysis was conducted specifically on patients who exhibited suicidal ideation at the beginning of the study. Results indicated that the combination of venlafaxine and aripiprazole led to a higher rate of resolution of suicidal ideation compared to placebo. Among participants with suicidal ideation at baseline (30 out of 91 on aripiprazole and 25 out of 90 on placebo), it resolved in 22 out of 30 (73.3%) in the aripiprazole group versus 11 out of 25 (44.0%) in the placebo group. It is important to note that patients with suicidal ideation represented a smaller subset of the original study population, which could affect the precision of the subgroup analysis^86^.

According to the 2019 Department of Veterans Affairs Department of Defense (VA/DoD) Clinical Practice Guideline for the Assessment and Management of Patients at Risk for Suicide, several recommendations are proposed for managing suicidal ideation across different psychiatric conditions. For patients presenting with suicidal ideation alongside major depressive disorder, the guideline suggests considering ketamine infusion as an adjunctive treatment to achieve short-term reduction in suicidal thoughts. For individuals diagnosed with bipolar disorder, the guideline recommends the use of lithium either as a standalone treatment or in combination with another psychotropic agent to mitigate the risk of suicide. Furthermore, among patients with schizophrenia or schizoaffective disorder who exhibit suicidal ideation or have a history of suicide attempts, the guideline advises the use of clozapine to lower the risk of death by suicide^87^.

Wilkinson and colleagues conducted a systematic review and individual participant data meta-analysis in 2018 to examine the impact of a single intravenous dose of ketamine on suicidal ideation. Their findings demonstrated that ketamine rapidly alleviated suicidal thoughts within 24 hours and maintained this effect for up to one week in depressed patients with suicidal ideation. Furthermore, they observed that ketamine's efficacy in reducing suicidal ideation was partially distinct from its effects on mood. However, the authors emphasize the necessity of subsequent trials in transdiagnostic populations to verify if ketamine exerts a specific effect on suicidal ideation. They also stress the importance of further research into ketamine's long-term safety and effectiveness^88^. In the 2024 systematic review and meta-analysis, Wang et al. comprehensively examined the efficacy of esketamine and ketamine in reducing SI among individuals with treatment-resistant depression (TRD). Their findings revealed that neither esketamine nor ketamine demonstrated a significant reduction in SI when compared to a placebo group. This conclusion underscores the necessity for further investigation to ascertain the true effects of these substances on suicidal ideation in TRD patients^89^.

One study conducted by A. Nierenberg and colleagues aimed to investigate whether a fixed-dose combination of oral D-cycloserine (DCS) and lurasidone (NRX-101) could better maintain improvement compared to lurasidone alone, following initial improvement with intravenous ketamine in patients with bipolar disorder experiencing severe depression and acute suicidal thoughts or behavior. The study, conducted as a multi-center, double-blind, two-stage, parallel randomized trial, enrolled 22 adult patients with bipolar disorder who were initially infused with either ketamine or saline. Subsequently, those who showed improvement were randomized into Stage 2, where they received either the fixed-dose combination of DCS and lurasidone or lurasidone alone. Results revealed that the DCS and lurasidone combination was significantly more effective in maintaining improvements in depression and reducing suicidal ideation compared to lurasidone alone. Moreover, this treatment regimen did not lead to significant safety concerns and showed improvements in patient- reported side effects. This study provides promising insights into potential treatment approaches for severe bipolar depression with acute suicidal ideation and behavior^90^.

More recent studies focus on newer potential drugs. Animal studies suggest that ebselen, akin to lithium, inhibits IMPase and elicits neuro-pharmacological effects on serotonin systems comparable to those of lithium. Additionally, ebselen demonstrates the ability to reduce motor impulsivity in animal models. Given the role of the serotonin system in mediating the antidepressant effects of lithium in TRD, this indicates the potential utility of ebselen for this condition. In human subjects, therapeutic doses of ebselen lead to reduced brain inositol levels, implying engagement with IMPase. However, ebselen also attenuates glutamate cycling, which could influence its antidepressant and neuroprotective properties. Neuropsychological investigations into emotional processing and reward- seeking further support the potential antidepressant efficacy of ebselen. Notably, ebselen diminishes impulsivity in human laboratory settings, raising the possibility, similar to lithium, of its utility in preventing suicide in mood disorder patients and mitigating impulsive behavior in other significant conditions like pathological gambling^91^. Victoria C. de Leon and colleagues published a brief report in 2023 regarding the current knowledge regarding nitrous oxide (N2O), a gas commonly used as an anesthetic. It has so far demonstrated rapid antidepressant effects similar to ketamine, and its potential in reducing SI is currently under investigation. Results showed a significant reduction in suicidal ideation with N2O compared to placebo at 24 hours post-inhalation, suggesting its potential for rapid anti-suicidal effects in depressed individuals. Further research is needed to explore the persistence of this effect^92^.

### 6.3. Non-pharmacological treatment

Non-pharmacological treatment includes a series of psychological interventions like cognitive-behavioral therapy (CBT) or dialectical behavior therapy (DBT). Psychotherapy can be individual, in small groups or in community settings. Because suicide is a complex phenomenon that, in most cases, occurs in the context of another psychiatric diagnosis, most of the recommendations are made for the mental health problem that resides. CBT can reduce suicide attempts, suicidal ideation and hopelessness but does not seem to prevent or reduce suicide. DBT combines elements of CBT, skills training, and mindfulness techniques with the aim of helping persons with BPD develop skills in emotional regulation, interpersonal effectiveness, and distress tolerance^93-95^.

The management of non-pharmacological suicide entails a nuanced array of psychotherapeutic modalities, with particular attention to addressing concurrent psychiatric conditions such as depression and anxiety. Cognitive-behavioral therapy, dialectical behavior therapy, interpersonal therapy, and psychodynamic therapy emerge as pivotal interventions, each characterized by distinct session frequencies and durations. The European Psychiatric Association underscores the significance of optimizing the therapeutic frequency and duration, advocating for a weekly session regimen, while the World Health Organization accentuates the therapeutic efficacy of psychotherapy, notably emphasizing CBT, behavioral activation, interpersonal therapy, and problem-solving therapy for depression and anxiety. The integration of follow- up sessions stands as a cornerstone strategy to avert relapse. Notably, individualized patient preferences and clinical exigencies warrant meticulous consideration in the selection and tailoring of psychotherapeutic interventions ^6,96-98^ (Table 2).

**Table 2:** Table 2. Psychotherapy recommendations for mental health disorders associated with high risk of suicidality

A	Type of psychotherapy	Frequency recommended
American Psychiatric Association^96^	Behavioral therapy	20 to 24 sessions with a recurrence of once a week
	Cognitive interventions	28 sessions with a recurrence of once a week
	CBT	between 6 and 20 sessions with a recurrence of once a week
	Interpersonal therapy	between 16 and 20 sessions with a recurrence of once a week
	Cognitive intervention	weekly group sessions and additional booster sessions after
	based on mindfulness	
	Psychodynamic therapy	between 3 and 80 sessions with a recurrence of once a week
	Supportive therapy	between 4 and 20 sessions with a recurrence of once a week
		or one every two weeks
The National Institute for Health and Care Excellence from the United Kingdom (NICE)^97^	CBT	8 sessions with a recurrence of once a week
	Behavioral activation	8 sessions with a recurrence of once a week (there may be more needed)
	Interpersonal therapy	between 8 and 16 sessions with a recurrence of once a week (there may be more needed)
	Psychodynamic therapy	between 8 and 16 sessions with a recurrence of once a week (there may be more needed)
	Group therapy	Group exercises – 10 sessions with a recurrence of once a week Mindfulness – 8 sessions with a recurrence of once a week Group CBT – 8 sessions with a recurrence of once a week Group behavioural activation – 8 sessions with a recurrence of once a week
European Psychiatric Association (EPA)^98^	EPA states that the efficacy of psychotherapy in depressive disorders is deemed to be correlated with the “dose” of therapy, specifically the number of sessions provided within a certain timeframe (evidence level: 1+; recommendation grade: A). Psychotherapy should be administered in the acute phase and should possess appropriate duration and frequency. Sessions ought to occur at least once per week (evidence level: 4, recommendation grade: Good Practice Point, GPP). To prevent relapse, psychotherapeutic interventions should encompass follow-up sessions (evidence level: 4, recommendation grade: GPP).	
World Health Organisation (WHO)^6^	Behavioural activation, CBT, interpersonal therapy and problem-solving therapy are recommended.	

## 7. CONCLUSION

In conclusion, the phenomenon of suicide stands as a poignant testament to the intricate interplay between individual psychosocial factors and broader societal dynamics. Its profound impact reverberates across cultures and generations, posing significant challenges to mental health professionals, policymakers, and society as a whole. Despite centuries of inquiry and intervention, suicide continues to take a heavy toll on communities worldwide, highlighting the imperative for ongoing research, prevention efforts, and compassionate care. The evolution of understanding surrounding suicide - from ancient perceptions rooted in moral judgment to contemporary insights informed by scientific inquiry - underscores the complexity of this issue and the need for nuanced, multifaceted approaches to address it effectively. While strides have been made in identifying risk and protective factors, enhancing clinical assessment, and implementing prevention strategies, much work remains to be done. It is incumbent upon us as a global community to prioritize suicide prevention, destigmatize mental health discourse, and foster supportive environments where individuals in distress feel empowered to seek help. By embracing a holistic approach that integrates psychosocial support, evidence-based interventions, and community engagement, we can strive towards a future where the tragedy of suicide is increasingly rare and every individual feels valued, supported, and connected. In this pursuit, may empathy, resilience, and solidarity guide our collective efforts to confront and ultimately overcome the scourge of suicide.

## Bullet Points

*· There is a strong association between suicide and mental health disorders*

*· Suicide prevention requires a multifaceted approach that includes both pharmacological and non- pharmacological interventions. Pharmacotherapy plays a crucial role in reducing suicidal ideation and behavior. Non-pharmacological treatments are essential components of suicide prevention strategies*

*· Addressing the complex challenges of suicide prevention requires collaborative efforts involving mental health professionals, policymakers, and society as a whole*

*· These efforts should focus on destigmatizing mental health discourse, enhancing clinical assessment, and fostering supportive environments where*

## References

[1] Isometsä Erkki (2014). Suicidal Behaviour in Mood Disorders—Who, When, and Why?. The Canadian Journal of Psychiatry.

[2] The Lancet Psychiatry (2020). Suicide and the psychiatrist. The Lancet Psychiatry.

[3] Naghavi Mohsen (2019). Global, regional, and national burden of suicide mortality 1990 to 2016: systematic analysis for the Global Burden of Disease Study 2016. BMJ.

[4] Yuodelis‐Flores Christine, Ries Richard K. (2015). Addiction and suicide: A review. The American Journal on Addictions.

[5] Zalsman Gil, Hawton Keith, Wasserman Danuta, van Heeringen Kees, Arensman Ella, Sarchiapone Marco, Carli Vladimir, Höschl Cyril, Barzilay Ran, Balazs Judit, Purebl György, Kahn Jean Pierre, Sáiz Pilar Alejandra, Lipsicas Cendrine Bursztein, Bobes Julio, Cozman Doina, Hegerl Ulrich, Zohar Joseph (2016). Suicide prevention strategies revisited: 10-year systematic review. The Lancet Psychiatry.

[6] (2021). Organization. WHO.int [Online].; 2021 [cited 2024 May 20th].

[7] Ougrin Dennis, Tranah Troy, Stahl Daniel, Moran Paul, Asarnow Joan Rosenbaum (2015). Therapeutic Interventions for Suicide Attempts and Self-Harm in Adolescents: Systematic Review and Meta-Analysis. Journal of the American Academy of Child &amp; Adolescent Psychiatry.

[8] McLean J., Maxwell M., Platt S., Harris F., Jepson R. (2008). Risk and Protective Factors for Suicide and Suicidal Behaviour: A Literature Review. Scottish Government (Social Research).

[9] De Berardis Domenico, Martinotti Giovanni, Di Giannantonio Massimo (2018). Editorial: Understanding the Complex Phenomenon of Suicide: From Research to Clinical Practice. Frontiers in Psychiatry.

[10] Tondo L. (2014). Chapter 1 - Brief history of suicide in Western cultures.

[11] Lu Da-Yong, Wu Hong-Ying, Cao Shan, Che Jin-Yu (2020). Historical analysis of suicide. Journal of Translational Genetics and Genomics.

[12] Kapur NaRG (2019). A brief history of suicidal behaviour. In Suicide Prevention.

[13] Moore Matthew D. (2016). Durkheim's types of suicide and social capital: a cross‐national comparison of 53 countries. International Social Science Journal.

[14] (1898). Le Suicide: Étude de Sociologie. Par Emile Durkheim. Paris: Alcan, 1897. 8vo, pp. 462. Price 7 f. 50 c.. Journal of Mental Science.

[15] Mueller Anna S., Abrutyn Seth, Pescosolido Bernice, Diefendorf Sarah (2021). The Social Roots of Suicide: Theorizing How the External Social World Matters to Suicide and Suicide Prevention. Frontiers in Psychology.

[16] George Ritzer DJG (2004). Sociological theory Boston. McGraw-Hill; 2004.

[17] Lester D (1968). Henry and Short on suicide: a critique.. The Journal of psychology.

[18] Wray Matt, Colen Cynthia, Pescosolido Bernice (2011). The Sociology of Suicide. Annual Review of Sociology.

[19] Mann J. John, Rizk Mina M. (2020). A Brain-Centric Model of Suicidal Behavior. American Journal of Psychiatry.

[20] (2012). Stress–Diathesis Model of Suicidal Behavior. The Neurobiological Basis of Suicide.

[21] Mann J. John (2003). Neurobiology of suicidal behaviour. Nature Reviews Neuroscience.

[22] Carballo Juan J., Akamnonu Chibuikem P., Oquendo Maria A. (2008). Neurobiology of Suicidal Behavior. An Integration of Biological and Clinical Findings. Archives of Suicide Research.

[23] Bun-Hee L., Yong-Ku K. (2006). Neurobiological Factors Associated with Suicidal Behavior.. Psychiatric Investigations.

[24] (2021). Administration SSAaMHS. samsha.gov. [Online] [cited 2024 April 1st.].

[25] (2023). Suicide Data and Statistics. Center for Disease Control and Prevention [cited 2024, April 1st.].

[26] (2021). Suicidology. Suicidology; suicidology.org. [Online] [cited 2024 February 22nd].

[27] Ajdacic-Gross Vladeta (2008). Methods of suicide: international suicide patters derived from the WHO mortality database. Bulletin of the World Health Organization.

[28] Jordans Mark JD, Kaufman Anne, Brenman Natassia F, Adhikari Ramesh P, Luitel Nagendra P, Tol Wietse A, Komproe Ivan (2014). Suicide in South Asia: a scoping review. BMC Psychiatry.

[29] (2000). Suicide acts in 8 states: incidence and case fatality rates by demographics and method. American Journal of Public Health.

[30] Bachmann Silke (2018). Epidemiology of Suicide and the Psychiatric Perspective. International Journal of Environmental Research and Public Health.

[31] (2024). Statistical report: near to real-time suspected suicide surveillance (nRTSSS) for England for the 15 months to August 2023. Office of Health Improvements and Disparities; www.gov.uk [cited 2024, April 1st].

[32] O’Connor Daryl B., Gartland Nicola, O’Connor Rory C. (2020). Stress, cortisol and suicide risk. International Review of Neurobiology.

[33] Bernanke J A, Stanley B H, Oquendo M A (2017). Toward fine-grained phenotyping of suicidal behavior: the role of suicidal subtypes. Molecular Psychiatry.

[34] Klempan T A, Sequeira A, Canetti L, Lalovic A, Ernst C, ffrench-Mullen J, Turecki G (2007). Altered expression of genes involved in ATP biosynthesis and GABAergic neurotransmission in the ventral prefrontal cortex of suicides with and without major depression. Molecular Psychiatry.

[35] Sudol Katherin, Mann J. John (2017). Biomarkers of Suicide Attempt Behavior: Towards a Biological Model of Risk. Current Psychiatry Reports.

[36] Lewis Charles P., Port John D., Blacker Caren J., Sonmez A. Irem, Seewoo Bhedita J., Leffler Jarrod M., Frye Mark A., Croarkin Paul E. (2020). Altered anterior cingulate glutamatergic metabolism in depressed adolescents with current suicidal ideation. Translational Psychiatry.

[37] Wisłowska-Stanek Aleksandra, Kołosowska Karolina, Maciejak Piotr (2021). Neurobiological Basis of Increased Risk for Suicidal Behaviour. Cells.

[38] Erhardt Sophie, Lim Chai K, Linderholm Klas R, Janelidze Shorena, Lindqvist Daniel, Samuelsson Martin, Lundberg Kristina, Postolache Teodor T, Träskman-Bendz Lil, Guillemin Gilles J, Brundin Lena (2012). Connecting inflammation with glutamate agonism in suicidality. Neuropsychopharmacology.

[39] Brundin L, Sellgren C M, Lim C K, Grit J, Pålsson E, Landén M, Samuelsson M, Lundgren K, Brundin P, Fuchs D, Postolache T T, Traskman-Bendz L, Guillemin G J, Erhardt S (2016). An enzyme in the kynurenine pathway that governs vulnerability to suicidal behavior by regulating excitotoxicity and neuroinflammation. Translational Psychiatry.

[40] Mann J. John (2013). The serotonergic system in mood disorders and suicidal behaviour. Philosophical Transactions of the Royal Society B: Biological Sciences.

[41] Oquendo Maria A., Sullivan Gregory M., Sudol Katherin, Baca-Garcia Enrique, Stanley Barbara H., Sublette M. Elizabeth, Mann J. John (2014). Toward a Biosignature for Suicide. American Journal of Psychiatry.

[42] Berardelli Isabella, Serafini Gianluca, Cortese Natalia, Fiaschè Federica, O’Connor Rory C, Pompili Maurizio (2020). The Involvement of Hypothalamus–Pituitary–Adrenal (HPA) Axis in Suicide Risk. Brain Sciences.

[43] Vargas-Medrano Javier, Diaz-Pacheco Valeria, Castaneda Christopher, Miranda-Arango Manuel, Longhurst Melanie O, Martin Sarah L., Ghumman Usman, Mangadu Thenral, Chheda Sadhana, Thompson Peter M., Gadad Bharathi S. (2020). Psychological and neurobiological aspects of suicide in adolescents: Current outlooks. Brain, Behavior, &amp; Immunity - Health.

[44] van Heeringen Kees, Mann J John (2014). The neurobiology of suicide. The Lancet Psychiatry.

[45] Bertolote José Manoel, Fleischmann Alexandra, De Leo Diego, Wasserman Danuta (2004). Psychiatric Diagnoses and Suicide: Revisiting the Evidence. Crisis.

[46] Bertolote José Manoel, Fleischmann Alexandra, De Leo Diego, Wasserman Danuta (2003). Suicide and mental disorders: do we know enough?. British Journal of Psychiatry.

[47] Agolli Arjola, Agolli Olsi, Chowdhury Selia, Shet Vallabh, Benitez Johanna S. Canenguez, Bheemisetty Niharika, Waleed Madeeha Subhan (2022). Increased cannabis use in pregnant women during COVID-19 pandemic. Discoveries.

[48] Yan Yifei, Hou Jianhua, Li Qing, Yu Nancy Xiaonan (2023). Suicide before and during the COVID-19 Pandemic: A Systematic Review with Meta-Analysis. International Journal of Environmental Research and Public Health.

[49] Dubé Justin P., Smith Martin M., Sherry Simon B., Hewitt Paul L., Stewart Sherry H. (2021). Suicide behaviors during the COVID-19 pandemic: A meta-analysis of 54 studies. Psychiatry Research.

[50] Goldacre M, Seagroatt V, Hawton K (1993). Suicide after discharge from psychiatric inpatient care. The Lancet.

[51] Chung Daniel Thomas, Ryan Christopher James, Hadzi-Pavlovic Dusan, Singh Swaran Preet, Stanton Clive, Large Matthew Michael (2017). Suicide Rates After Discharge From Psychiatric Facilities. JAMA Psychiatry.

[52] Balhara YP., Verma R. (2012). Schizophrenia and suicide. East Asian Arch Psychiatry.

[53] Sher Leo, Kahn René S. (2019). Suicide in Schizophrenia: An Educational Overview. Medicina.

[54] Hawton Keith, Sutton Lesley, Haw Camilla, Sinclair Julia, Deeks Jonathan J. (2005). Schizophrenia and suicide: Systematic review of risk factors. British Journal of Psychiatry.

[55] Edwards Alexis C., Abrahamsson Linda, Crump Casey, Sundquist Jan, Sundquist Kristina, Kendler Kenneth S. (2024). Alcohol use disorder and risk of specific methods of suicide death in a national cohort. Acta Psychiatrica Scandinavica.

[56] Grande Iria, Berk Michael, Birmaher Boris, Vieta Eduard (2016). Bipolar disorder. The Lancet.

[57] Cipriani Andrea, Pretty Heather, Hawton Keith, Geddes John R. (2005). Lithium in the Prevention of Suicidal Behavior and All-Cause Mortality in Patients With Mood Disorders: A Systematic Review of Randomized Trials. American Journal of Psychiatry.

[58] Malhi Gin S, Bell Erica, Bassett Darryl, Boyce Philip, Bryant Richard, Hazell Philip, Hopwood Malcolm, Lyndon Bill, Mulder Roger, Porter Richard, Singh Ajeet B, Murray Greg (2020). The 2020 Royal Australian and New Zealand College of Psychiatrists clinical practice guidelines for mood disorders. Australian &amp; New Zealand Journal of Psychiatry.

[59] Håkansson Anders, Karlsson Anna (2020). Suicide Attempt in Patients With Gambling Disorder—Associations With Comorbidity Including Substance Use Disorders. Frontiers in Psychiatry.

[60] Komoto Yasunobo (2014). Factors Associated with Suicide and Bankruptcy in Japanese Pathological Gamblers. International Journal of Mental Health and Addiction.

[61] Doering S, Probert-Lindström S, Ehnvall A, Wiktorsson S, Öberg NP, Bergqvist E (2024). Anxiety symptoms preceding suicide: A Swedish nationwide record review. J Affect Disord..

[62] Plutchik Robert, van Praag Herman M., Conte Hope R., Picard Susan (1989). Correlates of suicide and violence risk 1: The suicide risk measure. Comprehensive Psychiatry.

[63] Plutchik Robert, van Praag Herman M., Conte Hope R. (1989). Correlates of suicide and violence risk: III. A two-stage model of countervailing forces. Psychiatry Research.

[64] Apter A., Plutchik R., van Praag H. M. (1993). Anxiety, impulsivity and depressed mood in relation to suicidal and violent behavior. Acta Psychiatrica Scandinavica.

[65] Barzilay Shira, Apter Alan (2014). Psychological Models of Suicide. Archives of Suicide Research.

[66] Mann J. John, Currier Dianne (2021). Biological predictors of suicidal behaviour in mood disorders. Oxford Textbook of Suicidology and Suicide Prevention.

[67] Whiteside Stephen P., Lynam Donald R. (2001). The Five Factor Model and impulsivity: using a structural model of personality to understand impulsivity. Personality and Individual Differences.

[68] Moore Fhionna R., Doughty Heather, Neumann Tabea, McClelland Heather, Allott Claire, O'Connor Rory C. (2022). Impulsivity, aggression, and suicidality relationship in adults: A systematic review and meta-analysis. eClinicalMedicine.

[69] Paris Joel (2004). Half in Love with Easeful Death: The Meaning of Chronic Suicidality in Borderline Personality Disorder. Harvard Review of Psychiatry.

[70] Goodman Marianne, Tomas Irene Alvarez, Temes Christina M., Fitzmaurice Garrett M., Aguirre Blaise A., Zanarini Mary C. (2017). Suicide attempts and self‐injurious behaviours in adolescent and adult patients with borderline personality disorder. Personality and Mental Health.

[71] Joiner Thomas E., Brown Jessica S., Wingate LaRicka R. (2005). The Psychology and Neurobiology of Suicidal Behavior. Annual Review of Psychology.

[72] Newhill Christina E., Eack Shaun M., Mulvey Edward P. (2009). Violent Behavior in Borderline Personality. Journal of Personality Disorders.

[73] McGirr Alexander, Paris Joel, Lesage Alain, Phil. M., Renaud Johanne, Turecki Gustavo (2007). Risk Factors for Suicide Completion in Borderline Personality Disorder. The Journal of Clinical Psychiatry.

[74] Brodsky Beth S., Groves Shelly A., Oquendo Maria A., Mann J. John, Stanley Barbara (2006). Interpersonal Precipitants and Suicide Attempts in Borderline Personality Disorder. Suicide and Life-Threatening Behavior.

[75] (2024). Ask Suicide-Screening Questions (ASQ) Toolkit.. NIH-NIMH [cited 2024 February 21st].

[76] Horowitz Lisa M., Bridge Jeffrey A., Teach Stephen J., Ballard Elizabeth, Klima Jennifer, Rosenstein Donald L., Wharff Elizabeth A., Ginnis Katherine, Cannon Elizabeth, Joshi Paramjit, Pao Maryland (2012). Ask Suicide-Screening Questions (ASQ). Archives of Pediatrics &amp; Adolescent Medicine.

[77] (2020). Suicide prevention – the role of psychiatry [Online].; 2020 .. Psychiatrists RANZCP [cited 2024 January 15th].

[78] (2003). Practice guideline for the assessment and treatment of patients with suicidal behaviors.. The American journal of psychiatry.

[79] (2022). Preventing Suicide; 2022. CDC [cited 2024 February 22nd].

[80] Mann J John, Apter Alan, Bertolote Jose, Beautrais Annette, Currier Dianne, Haas Ann, Hegerl Ulrich, Lonnqvist Jouko, Malone Kevin, Marusic Andrej, Mehlum Lars, Patton George, Phillips Michael, Rutz Wolfgang, Rihmer Zoltan, Schmidtke Armin, Shaffer David, Silverman Morton, Takahashi Yoshitomo, Varnik Airi, Wasserman Danuta, Yip Paul, Hendin Herbert (2005). Suicide prevention strategies: a systematic review.. JAMA.

[81] Yao Zhixing, McCall William V (2023). Designing Clinical Trials to Assess the Impact of Pharmacological Treatment for Suicidal Ideation/Behavior: Issues and Potential Solutions.. Pharmaceutical medicine.

[82] (2010). PRACTICE GUIDELINE FOR THE Assessment and Treatment of Patients With Suicidal Behaviors. Association AP. psychiatryonline.org [cited 2024 Feb 22nd].

[83] Wasserman D, Rihmer Z, Rujescu D, Sarchiapone M, Sokolowski M, Titelman D, et al (2012). Az Európai Pszichiátriai Szövetség (European Psychiatric Association, EPA) úmutatója az öngyilkosság kezelésére és megelözésére [The European Psychiatric Association (EPA) guidance on suicide treatment and prevention].. Neuropsychopharmacol Hung..

[84] Masdrakis Vasilios G., Baldwin David S. (2023). Prevention of suicide by clozapine in mental disorders: systematic review. European Neuropsychopharmacology.

[85] Reeves Hollis, Batra Sachin, May Roberta S., Zhang Rusheng, Dahl Daniel C., Li Xiaohua (2008). Efficacy of Risperidone Augmentation to Antidepressants in the Management of Suicidality in Major Depressive Disorder. The Journal of Clinical Psychiatry.

[86] Lenze Eric J, Mulsant Benoit H, Blumberger Daniel M, Karp Jordan F, Newcomer John W, Anderson Stewart J, Dew Mary Amanda, Butters Meryl A, Stack Jacqueline A, Begley Amy E, Reynolds Charles F (2015). Efficacy, safety, and tolerability of augmentation pharmacotherapy with aripiprazole for treatment-resistant depression in late life: a randomised, double-blind, placebo-controlled trial. The Lancet.

[87] (2019). VA/DoD clinical practice guideline for the assessment and management of patients at risk for suicide. Department of Veterans Affairs DoD; www.healthquality.va.gov [cited 2024 March 20].

[88] Wilkinson Samuel T., Ballard Elizabeth D., Bloch Michael H., Mathew Sanjay J., Murrough James W., Feder Adriana, Sos Peter, Wang Gang, Zarate Carlos A., Sanacora Gerard (2018). The Effect of a Single Dose of Intravenous Ketamine on Suicidal Ideation: A Systematic Review and Individual Participant Data Meta-Analysis. American Journal of Psychiatry.

[89] Wang Ya-Ting, Wang Xiao-Le, Lei Lan, Guo Zhen-Yu, Kan Fei-Fei, Hu Die, Gai Cong, Zhang Yi (2023). A systematic review and meta-analysis of the efficacy of ketamine and esketamine on suicidal ideation in treatment-resistant depression. European Journal of Clinical Pharmacology.

[90] Nierenberg Andrew, Lavin Philip, Javitt Daniel C., Shelton Richard, Sapko Michael T., Mathew Sanjay, Besthof Robert E., Javitt Jonathan C. (2023). NRX-101 (D-cycloserine plus lurasidone) vs. lurasidone for the maintenance of initial stabilization after ketamine in patients with severe bipolar depression with acute suicidal ideation and behavior: a randomized prospective phase 2 trial. International Journal of Bipolar Disorders.

[91] Ramli Fitri Fareez, Cowen Philip J., Godlewska Beata R. (2022). The Potential Use of Ebselen in Treatment-Resistant Depression. Pharmaceuticals.

[92] Leon Victoria C. de, Kumar Arun, Nagele Peter, Palanca Ben J., Gott Britt, Janski Alvin, Zorumski Charles F., Conway Charles R. (2023). Nitrous Oxide Reduced Suicidal Ideation in Treatment-Resistant Major Depression in Exploratory Analysis. The Journal of Clinical Psychiatry.

[93] D'Anci Kristen E., Uhl Stacey, Giradi Gina, Martin Constance (2019). Treatments for the Prevention and Management of Suicide. Annals of Internal Medicine.

[94] Abdollahi Abbas, LeBouthillier Daniel M., Najafi Mahmoud, Asmundson Gordon J.G., Hosseinian Simin, Shahidi Shahriar, Carlbring Per, Kalhori Atefeh, Sadeghi Hassan, Jalili Marzieh (2017). Effect of exercise augmentation of cognitive behavioural therapy for the treatment of suicidal ideation and depression. Journal of Affective Disorders.

[95] Weitz Erica, Hollon Steven D., Kerkhof Ad, Cuijpers Pim (2014). Do depression treatments reduce suicidal ideation? The effects of CBT, IPT, pharmacotherapy, and placebo on suicidality. Journal of Affective Disorders.

[96] (2022). Clinical Practice Guideline for the Treatment of Depression Across Three Age Cohorts. APA; apa.org [cited 2024 February 22nd].

[97] (2024). NICE guidance. NICE (National Institute for Health and Care Excellence) [cited on 2024 January 15th].

[98] (2024). EPA Resource Center. EPA (European Psyhiatric Association [cited 2024 January 15th]..

